# Is MicroRNA-127 a Novel Biomarker for Acute Pancreatitis with Lung Injury?

**DOI:** 10.1155/2017/1204295

**Published:** 2017-12-24

**Authors:** Na Shi, Lihui Deng, Weiwei Chen, Xiaoxin Zhang, Ruijie Luo, Tao Jin, Yun Ma, Chen Du, Ziqi Lin, Kun Jiang, Jia Guo, Xiaonan Yang, Qing Xia

**Affiliations:** ^1^Department of Integrated Traditional Chinese and Western Medicine, West China Hospital of Sichuan University, Chengdu, China; ^2^Department of Gastroenterology, Northern Jiangsu People's Hospital, Yangzhou, Jiangsu Province, China; ^3^Department of Integrated Traditional Chinese and Western Medicine, People's Hospital of Wenjiang, Chengdu, China; ^4^Department of Science and Technology, West China Hospital of Sichuan University, Chengdu, China

## Abstract

**Background and Aims:**

The aim of this study was to determine the expression of microRNA-127 (miR-127) in both rat models and patients of acute pancreatitis (AP) with lung injury (LI).

**Methods:**

Rats were administrated with retrograde cholangiopancreatography injection of 0.5% or 3.5% sodium taurocholate to induce AP with mild or severe LI and were sacrificed at 6, 12, and 24 h. Rats from the control group received a laparotomy only. Plasma from a prospective cohort of AP patients was collected. The levels of miR-127 in the tissues and plasma were detected using quantitative reverse transcription-polymerase chain reaction (qRT-PCR).

**Results:**

The upregulation of miR-127 in the lungs of rats was detected in the groups of AP with severe LI at 6 h and 24 h, whereas it was scarcely detectable in plasma. In the pilot study that included 18 AP patients and 5 healthy volunteers, the plasma miR-127 level was significantly downregulated in AP patients with respiratory failure compared with the healthy volunteers (*P* = 0.014) and those without respiratory failure (*P* = 0.043).

**Conclusion:**

miR-127 might serve as a potential marker for the identification of AP with LI.

## 1. Introduction

Acute pancreatitis (AP) is a life-threatening inflammatory disease characterized by significant morbidity and mortality [1–3]. During the cascade events of activation of proinflammatory cytokines and mediators in AP, the injured cells of the lungs recruit immune cells and release cytokines, chemokines, growth factors, and prostaglandins, contributing to acute inflammatory response [4–7]. Lung injury (LI) is commonly involved in 15%~60% of severe AP, and severe LI is likely to trigger acute respiratory distress syndrome (ARDS) and respiratory failure (RF). When LI persists more than 48 h, it increases mortality of severer AP to 50% and accounts for 60% of mortality within the first week [8, 9]. Since its treatment is mainly supportive without targeted intervention to modify the progression, it is critical to identify those who are at risk for developing LI. Although the roles of cytokines and chemokines in respiratory complications of AP were proposed by extensive studies in the last two decades, the definite mechanism is yet to be fully elucidated [4]. Novel markers for early identification of AP with LI remain a great challenge.

MicroRNAs (miRNAs) are a category of small nonencoding RNAs of 22 nucleotides in length. They regulate gene expression through different mechanisms [10] and play a diverse role in many cellular processes [11–14]. The over- or underexpression of miRNAs is involved in various pathophysiological processes [15–18]. The discovery of misregulated miRNAs not only broadened our biological understanding of these diseases but also provided a new class of markers. An increasing number of evidence suggests that miRNAs may act as potential biomarkers for pancreatic injury [19–23]. An improvement in our understanding of the role that miRNAs play in AP may represent an attractive way to develop new diagnostic and prognostic tools for use in future clinical applications. Moreover, some miRNAs were reported to be correlated with the pathogenesis of lung diseases, such as lung cancer [24, 25], pulmonary fibrosis [26, 27], chronic obstructive pulmonary disease [28], and asthma [29, 30]. Here, microRNA-127 (miR-127) is one of the miRNAs that focuses on lung diseases [31–33]. miR-127 is originally found to be highly expressed in human and murine embryos and has a critical role in lung development and placental formation [34, 35]. Of note, recent study identified that miR-127 expression was aberrant in the inflammation-related pulmonary disorders [36] and further revealed that enhanced expression of miR-127 could promote the development of inflammatory macrophages and contribute to the exaggerated lung inflammation and injury [37].

Considering the potential role of miR-127 in the vital biological process of inflammation, we hypothesized that miR-127 may serve as a marker of AP with LI. To our best knowledge, it is the first study that integrated miR-127 and the inflammatory injuries of the pancreas and the lung. In this study, we sought to determine the expressions of miR-127 in the lung tissues of sodium taurocholate-induced AP models in rats and that in plasma of AP patients and to preliminarily explore the association of miR-217 levels and LI.

## 2. Materials and Methods

### 2.1. Animals

Fifty-four healthy male Sprague Dawley rats (weight, 250–300 g; age, 8–10 weeks) were purchased from Experiment Animal Center of Sichuan University. The rats were adaptively fed for one week prior to the experiments (maintained in a temperature-controlled room under a 12 h light/12 h dark cycle; fed with standard rat chow and tap water ad libitum). All animal experiments were undertaken in accordance with the National Institutes of Health Guide for the Care and Use of Laboratory Animals. The study protocols and experiments were approved by the Ethics Committee for Animal Experiments of Sichuan University.

### 2.2. Experimental Groups

Rats were fasted for 12 h and were given free access to tap water until 2 h prior to experiments, at which point they were randomized into the AP and control groups. Rat models of AP with mild LI (mLI) or severe LI (sLI) were induced by retrograde cholangiopancreatography injection of either 0.5% or 3.5% sodium taurocholate (0.1 ml/100 g body weight) at a rate of 0.1 ml/min using a micropump [38, 39]. Rats from the control group received a laparotomy only. All rats were subcutaneously rehydrated (2 mL per 100 g) after surgery and provided free access to water but were fasted after awakening from surgery. Rats were sacrificed at 6, 12, or 24 h after operation, which were termed AP-mLI 6, 12, and 24 h groups; AP-sLI 6, 12, and 24 h groups; and control 6, 12, and 24 h groups (*n* = 6 for each group).

### 2.3. Animal Sample Collection

Serum samples were collected and stored at −80°C. The right lung was placed in liquid nitrogen immediately after removal for the detection of myeloperoxidase (MPO) activity and miR-127 levels. The pancreas and left lung tissues were fixed in 10% neutral formalin for pathological hematoxylin and eosin (HE) staining. A blinded histological analysis of the pancreas [40, 41] and the lung [42, 43] was performed to evaluate the severity of tissue injury. For each pancreas pathological section, edema, inflammatory infiltration, and acinar cell necrosis were evaluated in 10 random visual fields under a 200x microscope (Table 1). A 4-point scale (0 = none, 1 = mild, 2 = moderate, and 3 = severe) was used to assess lung damage based on alveolar edema, inflammatory infiltration, and capillary congestion (Table 1). Serum amylase and inflammatory cytokine were determined according to instructions (amylase and MPO assay kit were provided by Nanjing Jiancheng Bioengineering Institute; Inflammatory cytokine ELISA kits were purchased from Xin Bo Sheng Biotechnology Co. Ltd.). The levels of miR-127 in serum and lung tissues were quantified using quantitative reverse transcription-polymerase chain reaction (qRT-PCR), and the expression between different time points was compared.

### 2.4. Patients

Plasma samples and clinical data from AP patients or healthy volunteers (HVs) were obtained from a prospective observational study that undertook measurement of various severity markers (including miRNA), in a range of patients with mild to severe AP. The study protocol and informed consent were approved by the Clinical Trials and Biomedical Ethics Committee of the West China Hospital of Sichuan University. All procedures in studies with human participants were performed in accordance with the ethical standards of the institutional and/or national research committee and with the 1964 Declaration of Helsinki as well as its later amendments or comparable ethical standards. All subjects provided written informed consent before enrolling.

### 2.5. Patient Criteria

The diagnosis of AP requires two of the following three features [44]: (1) abdominal pain consistent with AP; (2) serum amylase and/or lipase ≥ 3 times the upper limit of normal; and (3) characteristic findings of AP on CT scan, magnetic resonance imaging, or transabdominal ultrasonography. Mild AP was defined as the absence of both organ failure and local or systemic complications, moderately severe AP as the presence of transient organ failure (resolved within 48 h) or local/systemic complications in the absence of persistent organ failure (persists more than 48 h), and severe AP as persistent organ failure. In accordance with the modified Marshall scoring system, organ failure was defined as a score of 2 or more for one of three organ systems (respiratory, renal, and cardiovascular) [45].

AP patients between 18 and 70 years of age who were admitted to our hospital within 48 h after the onset of disease were enrolled. Patients were excluded if they (1) were pregnant or lactating, (2) had any malignant disease, or (3) had serious primary systemic diseases. HVs were sex- and age-matched for study patients.

A total of 23 subjects were selected. AP patients comprised two groups, the presence of respiratory failure (RF, *n* = 10) and nonrespiratory failure (nRF, *n* = 8), and were compared with HVs (*n* = 5). Demographics and clinical data of patients were recorded using uniform paper charts and electronic medical records, which were reviewed by 2 independent physicians.

### 2.6. Human Plasma Collection

Peripheral blood samples were collected from patients with AP using BD Vacutainer EDTA tubes within 24 h after admission. The processing of all blood samples began within 30 minutes after collection and was performed according to the following procedures: maintained upright for 20–25 minutes, centrifugation at room temperature (22–24°C) at 600*g* for 30 minutes, further centrifugation of the supernatant at 24°C and 1500*g* for 10 minutes, and storage of the supernatant at −80°C.

### 2.7. Quantitative Reverse Transcription-Polymerase Chain Reaction

Total RNA from lung tissues was extracted using TRIzol reagent. Total RNA was extracted from 100 *μ*l serum from rats or 400 *μ*l plasma from human subjects using the mirVana™ PARIS™ Kit (Ambion, USA) following the manufacturer's protocol.

QRT-PCR was performed to detect and quantify miR-127 with TaqMan Small RNA Assays (Applied Biosystems, USA). Reverse transcription was performed using TaqMan miRNA reverse transcription kit (Applied Biosystems, USA) according to the manufacturer's instructions. PCR amplification was performed using TaqMan Universal Master Mix (Applied Biosystems, USA) on an AB7900HT with cycling conditions as recommended by Applied Biosystems. Each reaction was performed in triplicates containing 0.75 *μ*l cDNA, 0.5 *μ*l 20 × TaqMan microRNA Assay Mix, 5 *μ*l 2 × TaqMan Universal PCR buffer, and 3.75 *μ*l ddH_2_O. The amplification was performed as follows: denaturation at 50°C for 2 min and at 95°C for 10 minutes, followed by 40 cycles of 95°C for 15 seconds and 60°C for 1 minute. miR-16 in plasma and U6 snRNA in the lung tissues were used as an internal control. The miRNA levels that were not detected after 45 cycles of real-time PCR were considered to have a threshold cycle (Ct) equivalent to 45.

### 2.8. Statistical Analysis

Statistical analyses were performed using SPSS 21.0 software. The Ct was defined as the fractional cycle number of fluorescence that passed through a given threshold. The target miRNA expression was analyzed by 2^−ΔCt^ (ΔCt = Ct_target miRNA_ − Ct_internal control_) using REST 2009 software (Qiagen Inc.). Continuous variables were expressed as the mean ± standard error, and categorical variables were expressed as proportions. One-way ANOVA, Kruskal-Wallis test, chi-square test, or Fisher's exact test were used, when appropriate, to determine significant differences between the groups. A two-sided *P* value less than 0.05 was considered statistically significant.

## 3. Results

### 3.1. Normal Markers in Serum and Tissues of Rats with AP-mLI and AP-sLI

**Figure 1:**
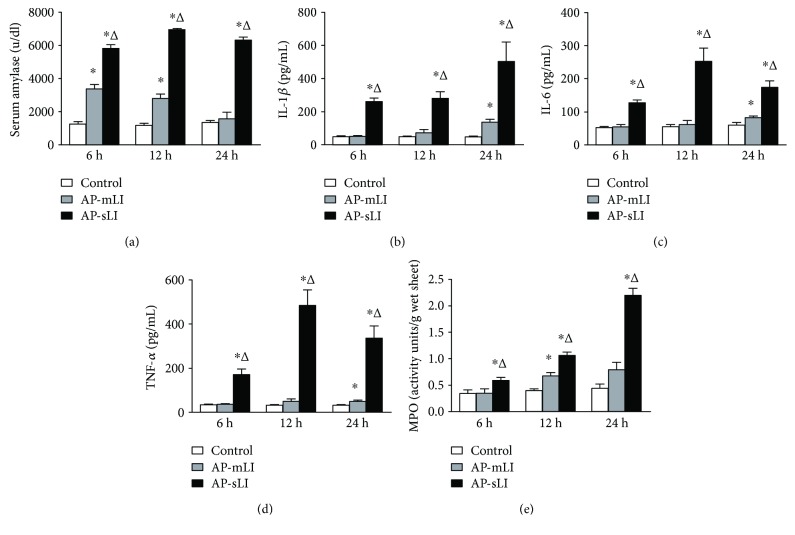
The levels of serum amylase (u/dl), serum cytokines, and MPO activity in the lung tissues in rats. (a) Serum amylase; (b) interleukin-1*β* (IL-1*β*); (c) interleukin-6 (IL-6); (d) tumor necrosis factor-*α* (TNF-*α*); and (f) myeloperoxidase (MPO). (mean with SEM, *N* = 6 for each group) ^∗^*P* < 0.05 versus control group; ^Δ^*P* < 0.05 versus AP-mLI group.

The levels of serum amylase (u/dl) in rats with AP were significantly increased compared to those in the control rats at each time point (Figure 1(a)). Serum amylase levels were significantly higher in the AP-mLI 6 and 12 h groups than in the control group and nearly returned to a normal level at 24 h. They were significantly elevated in the AP-sLI 6, 12, and 24 h groups than in both AP-mLI and control groups at each time point (all *P* < 0.05).

The levels of serum IL-1*β*, IL-6, and TNF-*α* were significantly elevated in AP-mLI group at 24 h than in the control group, and they were all significantly higher in the AP-sLI 6, 12, and 24 h groups than in both control and AP-mLI groups at each time point (all *P* < 0.05) (Figures 1(b), 1(c), and 1(d)).

As shown in Figure 1(e), MPO activity of the lung tissues in AP-mLI group was significantly higher than that in the control group at 12 h (*P* < 0.05), and no significant difference was found at 6 and 24 h. In AP-sLI group, MPO activity of the lung tissues was significantly higher than those in both control and AP-mLI groups at each time point (all *P* < 0.05).

### 3.2. Histopathologic Severity of the Pancreas and the Lung of Rats with AP-mLI and AP-sLI

**Figure 2:**
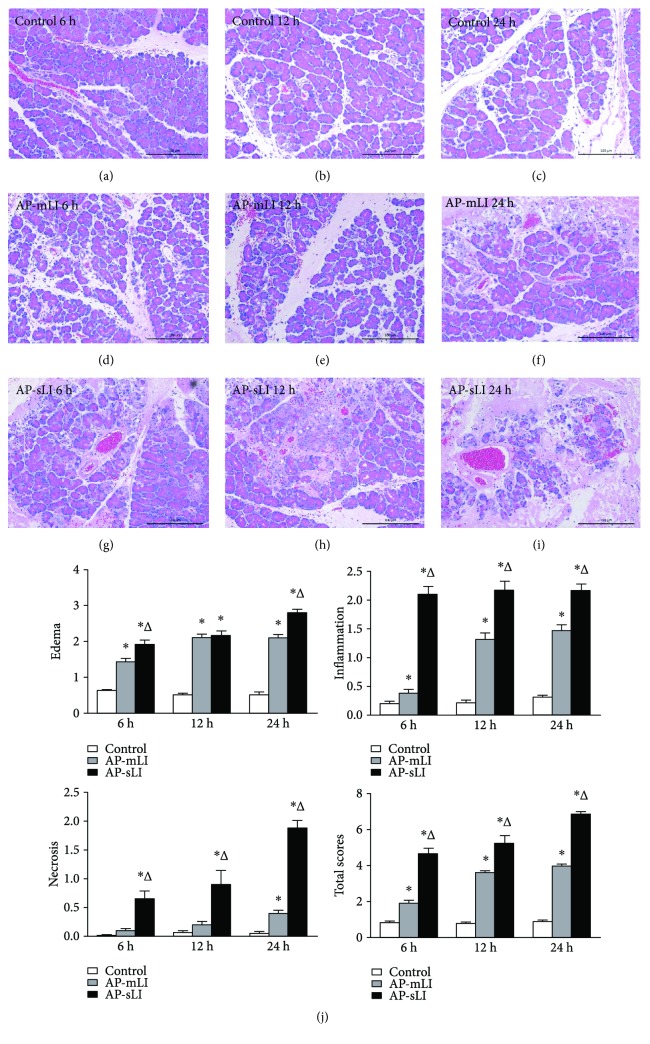
Histopathological changes of the pancreatic tissues. Light micrographs (×200) of the pancreas in the control rats at 6 h (a), 12 h (b), and 24 h (c), AP-mLI rats at 6 h (d), 12 h (e), and 24 h (f), and AP-sLI rats at 6 h (g), 12 h (h), and 24 h (i). (j) Blinded histopathological analysis for edema, inflammatory infiltration, acinar cellular necrosis, and total histological scores. ^∗^*P* < 0.05 versus control group; ^Δ^*P* < 0.05 versus AP-mLI group.

The histopathological changes in the pancreatic tissues are shown in Figure 2. The pancreatic tissues in the control group lacked obvious changes (Figures 2(a), 2(b), and 2(c)). Interstitial edema, inflammatory cell infiltration, and acinar cellular necrosis that are indicative of AP were seen in light micrographs (Figures 2(d), 2(e), 2(f), 2(g), 2(h), and 2(i)). Higher histopathologic scores were shown in both AP-mLI and AP-sLI groups at 6, 12, and 24 h after sodium taurocholate administration than in the control group at each time point (Figure 2(j)). Furthermore, the AP-sLI group exhibited more severe pancreatic injury than AP-mLI and control groups (all *P* < 0.05).

**Figure 3:**
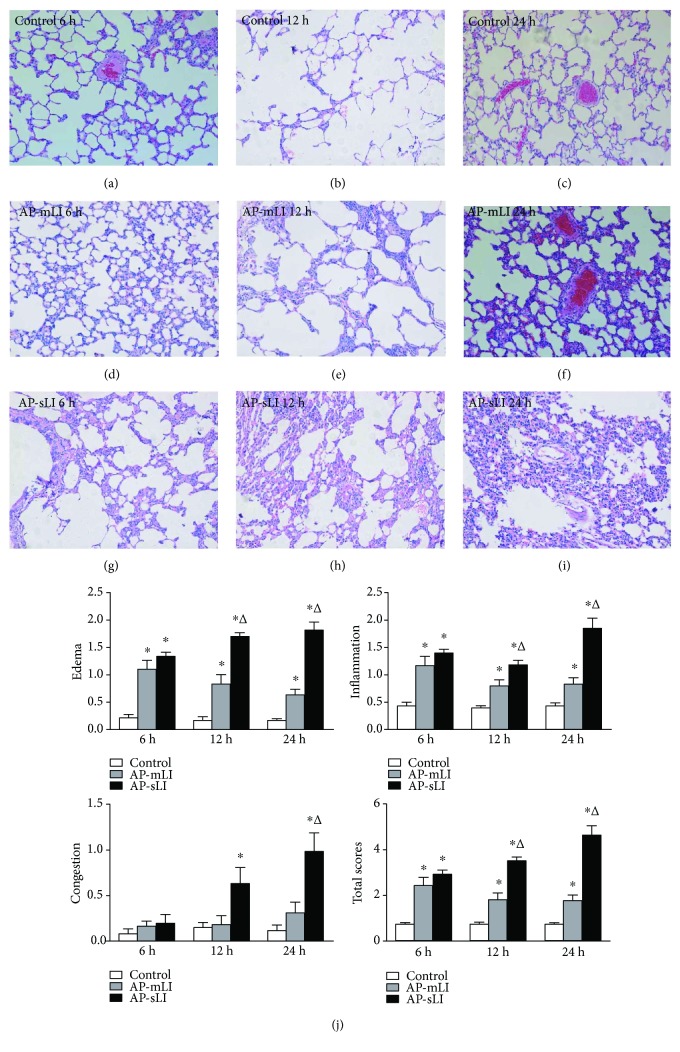
Histopathological changes of the lung tissues. Light micrographs (×200) of the lungs in the control rats at 6 h (a), 12 h (b), and 24 h (c), AP-mLI rats at 6 h (d), 12 h (e), and 24 h (f), and AP-sLI rats at 6 h (g), 12 h (h), and 24 h (i). (j) Blinded histopathological analysis for alveolar edema, inflammatory infiltration, capillary congestion, and total histological scores. ^∗^*P* < 0.05 versus control group; ^Δ^*P* < 0.05 versus AP-mLI group.

The histopathological changes in the lung tissues are shown in Figure 3. Rats in the control group exhibited no obvious changes (Figures 3(a), 3(b), and 3(c)). Interstitial edema, inflammatory cell infiltration, and widened alveolar septum in the lung tissue were observed in the AP-mLI group (Figures 3(d), 3(e), and 3(f)). Rats in the AP-sLI group exhibited a variety degree of alveolar and interstitial edema, hemorrhaging with atelectasis, widened alveolar septum, and the infiltration of inflammatory cells and red blood cells that are indicative of obvious LI (Figures 3(g), 3(h), and 3(i)). As shown in Figure 3(j), pathologic severity scores of the lung tissues were higher in both AP-mLI and AP-sLI groups than the control group at 6, 12, and 24 h. The AP-sLI group exhibited higher severity scores of the lungs than the AP-mLI group at 12 and 24 h (all *P* < 0.05).

### 3.3. miR-127 Was Significantly Upregulated in Lung Tissues of AP Rats

**Figure 4:**
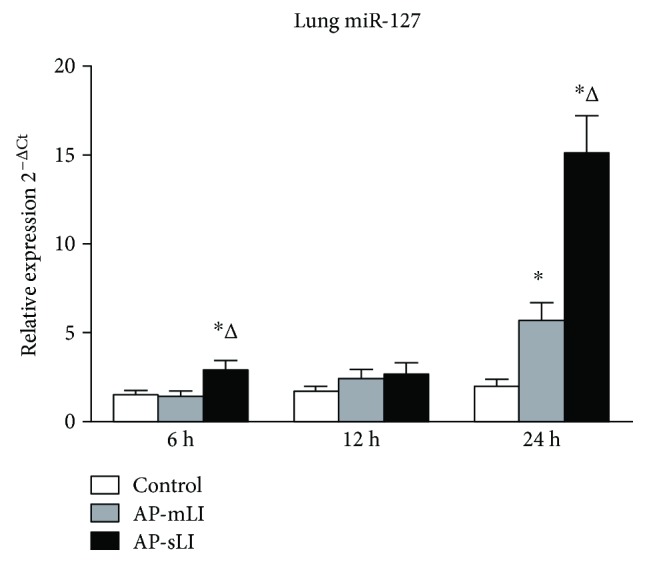
The relative expression of miR-127 in the lung tissues of rats. The level of miR-127 was expressed as 2^−ΔCt^ (ΔCt = Ct_miR-127_ − Ct_U6 snRNA_) using REST 2009 software (Qiagen Inc.). ^∗^*P* < 0.05 versus control group; ^Δ^*P* < 0.05 versus AP-mLI group.

The expression levels of miR-127 in the lung tissues of the rats in AP-mLI group were significantly higher than those in the control group at 24 h (Figure 4, *P* < 0.05) and were insignificantly different at 6 and 12 h (all *P* > 0.05). The upregulation of miR-127 in the AP-sLI group was significant than that in both AP-mLI and control groups at 6 and 24 h (*P* < 0.05). The levels of miR-127 in AP-mLI and AP-sLI groups at 12 h were slightly increased, but they did not significantly differ from those in the control group. No difference was observed in the expression level of miR-127 between AP-mLI and AP-sLI groups at 12 h. U6 snRNA in the lung tissues was insignificantly different in any of the groups.

### 3.4. miR-127 Was Lowly Expressed in Serum of Rats

We determined the expression levels of miR-127 in serum of the rats using miR-16 as an internal control. The expression of serum miR-16 was successfully detected, and no significant difference was observed among the AP-mLI, AP-sLI, and control groups. However, miR-127 could not be detected by qRT-PCR in serum samples of the rats even with the modifications to the methods allowed their reliable quantitation (all nearly Ct = 45).

### 3.5. miR-127 Levels Positively Correlated with Pathological Severity in the Lung Tissues of Rats

A Pearson's correlation coefficient analysis showed that the miR-127 levels in the lung tissues were significantly positively correlated with pancreatic edema, inflammatory infiltration, acinar cell necrosis, and total scores (Table 2, all *P* < 0.05). The levels of lung miR-127 also demonstrated a significant positive correlation with lung edema, inflammatory infiltration, capillary congestion, and total scores (Table 2, all *P* < 0.05).

### 3.6. miR-127 in the Plasma of AP Patients and Healthy Controls

The age, body mass index (BMI), and routine biochemical parameters (including amylase and lipase) were similar between RF and nRF groups (all *P* > 0.05). In terms of single markers on admission, the patients in the RF group exhibited significantly lower oxygenation index (PaO_2_/FiO_2_), and higher C-reaction protein (CRP) and interleukin-6 (IL-6), than those in the nRF group. The modified Marshall scores in the RF group were significantly higher than those in the nRF group (2.40 ± 0.70 versus 1.13 ± 1.25; *P* = 0.014). All patients survived, and those who developed RF had a higher rate of infection and necrosis and longer length of hospital stay.

**Figure 5:**
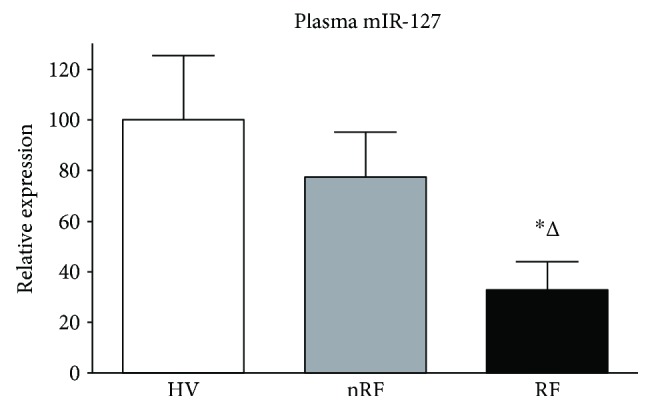
The relative expression of plasma miR-127 in the patients with AP and HVs. The level of miR-127 was expressed as 2^−ΔCt^ (ΔCt = Ct_miR-127_ − Ct_miR-16_) using REST 2009 software (Qiagen Inc.). Normalized HV group as 100. ^∗^*P* = 0.014 versus HVs; ^Δ^*P* = 0.043 versus nRF group.

The expression of miR-127 was successfully detected in plasma of both AP patients and the HVs (Figure 5). Similar expressions were detected in terms of plasma miR-127 between nRF and HV groups. Significant downregulation of plasma miR-127 was shown in the RF group than in the HV group (*P* = 0.014) and the nRF group (*P* = 0.043). No detectable difference was shown in the expression of plasma miR-16 as an internal control between AP patients and HVs.

## 4. Discussion

The identification of LI in AP patients for the potential improvement of the outcome remains a challenging issue. The discovery of miRNAs [46] provided a new class of disease markers. Studies in recent years have revealed that miRNAs might be novel biomarkers for AP. miR-127 has been reported to play a pivotal role in organic development [35, 47], ischemia/reperfusion injury [48], cancer [49], lung disease [37], and so forth. Nevertheless, the expressions of miR-127 and its role in AP with LI are unknown. This study explored the relationship between miR-127 and AP with LI from the perspective of blood and tissue.

In this study, retrograde cholangiopancreatography injection of sodium taurocholate was used to induce a rat model with AP complicated by LI, which is widely accepted by investigators of pancreatic diseases. Various degrees of injury in the pancreas and the lung could be achieved by adjustments of the dose and concentration of sodium taurocholate, rate of administration, and perfusion pressure by a syringe pump [50]. Based on the previous reports and our preliminary experiments, we used two concentration and dosages of sodium taurocholate for retrograde infusion. The results of biochemical tests and histopathological scoring of the pancreas and the lungs proved that sodium taurocholate successfully evoked a dose- and time course-dependent changes in terms of histopathological processes. Furthermore, higher dose of sodium taurocholate could induce more severe injuries with higher levels of serum IL-6, IL-1*β*, and TNF-*α* and MPO of the lungs. The histopathological scoring of the pancreas and the lungs showed that AP-mLI group exhibited obvious pancreatic injuries and mild LI, while AP-sLI group presented both more extensive and severe injuries in the pancreas and the lungs.

The levels of miR-127 were upregulated in the lung tissues of AP rats. Recent studies demonstrated the similar result that miR-127 was prominently induced in an exaggerated pulmonary inflammation and injury [32]. However, another study [37] showed that miR-127 was downregulated in lipopolysaccharide-stimulated LI, which appeared to be paradoxical to the previous study [32] and our findings. These results indicate the exact role of miR-127 in pulmonary inflammation, and injury is still controversial.

In this study, miR-127 levels presented a significant positive correlation with histopathological severity scores of the pancreas and the lungs. Furthermore, both miR-127 and proinflammatory cytokine (IL-1*β*, IL-6, and TNF-*α*) were increased in AP with LI. Although the mechanism involved has been largely undefined, the studies indicated that miR-127 promoted lung inflammation and injury by activating the inflammatory pathway [32]. Therefore, these results suggested that miR-127 in the lungs might reflect pancreatic and lung tissue injuries and play a potential role in the inflammatory signaling and lung pathology.

With regard to its potential as a marker for LI in AP, we verified its expressions in plasma of patients with AP-induced LI and HVs. The results showed that plasma miR-127 in the RF group tended to be significantly lower than those in the nRF group and the HV group. To the best of our knowledge, it was the first study reporting plasma miR-127 in AP patients. Among the limited studies on miR-127 in human pancreatic diseases, it was transiently expressed in pancreatic duct progenitor cells during the embryonic stages [51] and deregulated in pancreatic intraepithelial neoplasia [52] and in human pancreatic cancer [53]. Circulating miRNAs have been reported as promising novel noninvasive biomarkers in pathophysiological conditions, but little is known about the sources of circulating miRNAs and their relations with tissue miRNAs. Circulating miRNAs may be a result of the leakage of miRNAs into body fluid from tumor tissues or damaged tissues [54–56]. Furthermore, extensive studies in the last two decades have demonstrated that the first 24 h after the onset of symptoms are critical for identifying the at-risk group of patients and initiate aggressive treatment. Herein, we determined its expression in AP patients with early-stage AP. Thus, miR-127 might be a potential biomarker for early identification of RF in severe AP patients.

The major findings of this study were that miR-127 correlated to the severity of LI in AP rats and differentially expressed in plasma of AP patients with RF. Despite the potential interest in our findings, this study had limitations. First, miR-127 was undetectable in serum of rats, and the correlations of serum and tissue miR-127 were not analyzed. Second, miR-127 was detected in a small number of sample size (*n* = 23) for analysis in our study, and it was still lowly expressed. Third, pancreatitis is a complex and heterogeneous disease that evolves over time to time, and the discriminative ability of miR-127 at multiple time points should be determined in future studies.

## 5. Conclusions

miR-127 might be of help for the identification of AP with LI. A prospective, consecutive cohort study with a large sample is required to validate its value as a disease marker for AP in the future.

## Figures and Tables

**Table 1:** Histopathological scoring criteria of the pancreas and lung.

	Condition	Score	Indication
Pancreas	Edema	0	Absent
		1	Focally increased between lobules
		2	Diffusely increased between lobules
		3	Acini disrupted
		4	Acini separated
	Inflammatory cell infiltrate	0	Absent
		1	In ducts (around ductal margins)
		2	In the parenchyma (in <50% of the lobules)
		3	In the parenchyma (in 50%–75% of the lobules)
		4	In the parenchyma (in >75% of the lobules)
	Acinar necrosis	0	Absent
		1	Periductal necrosis (<5%)
		2	Focal parenchymal necrosis (5%–20%)
		3	Diffuse parenchymal necrosis (20%–50%)
		4	Diffuse parenchymal necrosis (>50%)

Lung	Alveolar edema	0	Absent
		1	Focally alveolar septum widened (<20%)
		2	Diffusely alveolar septum widened (20%–50%)
		3	Diffusely alveolar septum widened (>50%) with alveolar cell disrupted, separated
	Inflammatory infiltration	0	Absent
		1	In interstitial
		2	In alveoli and interstitial (<50%)
		3	In alveoli and interstitial (>50%)
	Capillary congestion	0	Absent
		1	Focally in alveoli and interstitial (<25%)
		2	Diffusely in alveoli and interstitial (25%–50%)
		3	Diffusely in alveoli and interstitial (>50%)

**Table 2:** The correlations of miR-127 levels and pathological severity in the lung tissues of rats.

	Histopathologic scores	Lung miR-127	
		*r*	*P*
Pancreas	Edema	0.571	<0.001
	Inflammatory infiltration	0.495	<0.001
	Acinar cellular necrosis	0.703	<0.001
	Total scores	0.627	<0.001

Lung	Edema	0.470	<0.001
	Inflammatory infiltration	0.662	<0.001
	Capillary congestion	0.637	<0.001
	Total scores	0.641	<0.001
